# Dr. John W. Cobb

**Published:** 1915-03

**Authors:** 


**Dr. John W. Cobb,** Albany, 1858; died at his home in Binghamton, January 14, of pneumonia, aged 76. He was a Civil War veteran.

